# The Physician’s Daily Pocket Record

**Published:** 1883-11

**Authors:** 


**The Physician's Daily Pocket Record,** comprising a Visiting List, many useful Memoranda Tables, etc. By S. W. BUTLER, M. D. Eighteenth year. New and thoroughly revised edition, with metric posological table. Edited by D. G. BURTON, M. D. Philadelphia: Published at the office of the *Medical and Surgical Reporter*, 115 South Seventh street. 1883.

"Butler's List" is well known and a favorite with a goodly proportion of the profession. It has an excellent and useful table showing the normal and abnormal qualities of urine, with their significance and tests. In its arrangements it has some advantages over other lists. It is also very compact and portable.

